# Actinobacteria Associated with the Marine Sponges *Cinachyra* sp., *Petrosia* sp., and *Ulosa* sp. and Their Culturability

**DOI:** 10.1264/jsme2.ME11270

**Published:** 2011-12-29

**Authors:** Shams Tabrez Khan, Motoki Takagi, Kazuo Shin-ya

**Affiliations:** 1 Biomedicinal Information Research Center (BIRC), Japan Biological Informatics Consortium (JBIC), 2–4–7 Aomi, Koto-ku, Tokyo 135–0064, Japan; 2 Biomedicinal Information Research Center (BIRC), National Institute of Advanced Industrial Science and Technology (AIST), 2–4–7 Aomi, Koto-ku, Tokyo 135–0064, Japan

**Keywords:** actinobacteria, marine sponge, *Cinachyra*, *Petrosia*, *Ulosa*

## Abstract

Actinobacteria associated with 3 marine sponges, *Cinachyra* sp., *Petrosia* sp., and *Ulosa* sp., were investigated. Analyses of 16S rRNA gene clone libraries revealed that actinobacterial diversity varied greatly and that *Ulosa* sp. was most diverse, while *Cinachyra* sp. was least diverse. Culture-based approaches failed to isolate actinobacteria from *Petrosia* sp. or *Ulosa* sp., but strains belonging to 10 different genera and 3 novel species were isolated from *Cinachyra* sp.

Actinobacteria isolated from terrestrial habitats are known to be the preeminent source of bioactive compounds. Recently, marine actinobacteria, especially those isolated from marine sponges, also have been identified as a rich source of bioactive metabolites (8, 17, 19). Although actinobacteria have been isolated from different marine habitats such as marine sediments, seawater, and marine invertebrates, marine sponges are of special interest, and many bioactive compounds isolated from marine sponges are already in clinical or preclinical trials (23). Bacteria associated with sponges are assumed to produce these compounds, as reported in the studies by Stierle *et al.*(28), Elyakov *et al.*(6), and Bewley *et al.*(4). This assumption is based on the following observations: (a) the correlation between the compounds produced by sponges and sponge taxonomy is weak; (b) distantly related sponges produce identical compounds; and (c) many compounds produced by sponges, such as peloruside A or laulimalide, are polyketides, which are a class of compounds produced by bacteria. Therefore, the study of microbial communities, especially actinobacteria, that are associated with different marine sponges is important in the search for novel compounds. Sponges can be divided into 2 groups based on the density of associated microorganisms (9): Sponges with a dense tissue matrix have low rates of water flowing through their bodies and therefore trap more bacteria; these sponges are referred to as high-microbial-abundance (HMA) sponges. In contrast, sponges with a more porous body matrix have higher rates of water flowing through their bodies and retain fewer bacteria in the mesophyl; these sponges are referred to as low-microbial-abundance (LMA) sponges. HMA sponges have been shown to harbor sponge-specific groups of bacteria, and LMA sponges have been shown to host few sponge-specific groups of bacteria (10, 31).

We recently isolated several actinobacteria from marine sponge samples (15, 17) collected mainly from Ishigaki Island, Okinawa Prefecture, Japan. These actinobacteria include phylogenetically new members of *Streptomyces*(16), a genus well known for the production of secondary metabolites. Many strains of this genus require salt for growth, indicating their marine origin. When screened for the production of bioactive metabolites, these strains have been found to produce new bioactive metabolites (13, 17, 30).

*Petrosia* sp. (*Demospongiae*, *Petrosiidae*), a common Mediterranean sponge, produces several bioactive polyacetylenes including acetylenic strongylodiols (20, 21, 35) and the steroid IPL-576,092, which is an antiasthmatic compound under clinical study (23). *Ulosa* sp. produces ulosantoin, which has insecticidal activity (32), and *Cinachyra* sp. produces the cytotoxic compound cinachyrolide A (7); however, to the best of our knowledge, the microbial communities associated with these sponges have not yet been studied. Only one report on the epibiotic bacteria of *Petrosia* sp. is available (5), and Vacelet and Donadey (31) found the cyanobacterium *Aphanocapsa feldmanni* in *Petrosia* sp., in specialized cells called bacteriocytes. Here, we describe actinobacteria associated with these 3 sponges using culture-dependent and -independent approaches.

*Cinachyra* sp., *Petrosia* sp., and *Ulosa* sp. were collected by scuba divers off the coast of Ishigaki Island, Okinawa Prefecture, Japan. DNAs were prepared using a Qiagen tissue kit (Qiagen, Valencia, CA, USA), and actinobacteria-specific primers S-C-Act-235-a-S-20 and S-C-Act-878-a-A-19, as described by Stach *et al.*(26), were used for 16S rRNA gene amplification. PCR products were cloned using a TOPO TA cloning kit (Invitrogen, Carlsbad, CA, USA) and sequenced with M13 primers in accordance with the manufacturer’s instructions. Sequence anomalies were verified using Pintail (1) and Bellerophon (http://comp-bio.anu.edu.au/bellerophon/bellerophon.pl, 11), available on the Green-genes website. Phylogenetic neighbors were searched using the BLAST program against the sequences available in the DNA Database of Japan (DDBJ). A total of 93, 63, and 81 clones from *Cinachyra* sp., *Petrosia* sp., and *Ulosa* sp., respectively, were sequenced. We used 98.5% of 16S rRNA gene sequence similarity as the cutoff value (27) for an operational taxonomic unit and the Fastgroup II online program (36) for analysis. The sequences from *Ulosa* sp. were dereplicated into 58 operational taxonomic units (OTUs), those from *Petrosia* sp. were dereplicated into 48 OTUs, and those from *Cinachyra* sp. were dereplicated into only 5 OTUs (Table 1). Rarefaction curves were also calculated using the Fastgroup II online program. The curves showed that actinobacterial diversity is comparable in *Petrosia* sp. and *Ulosa* sp. but is very low in *Cinachyra* sp. (data not shown). Based on the Shannon–Wiener index [*H′*= −∑*^S^_i_*_=1_ (*pi*ln*pi*)] and Simpson’s diversity index {*D*=∑ [*ni* (*ni*−1)/*N*(*N*−1)]} (12), actinobacterial diversity associated with these sponges was found to be distributed in the following order: *Ulosa* sp. > *Petrosia* sp. > *Cinachyra* sp. (Table 1).

Although actinobacteria-specific primers were used for 16S rRNA gene amplification, sequences belonging to other bacterial phyla were also recovered in these 16S rRNA gene libraries. In the *Ulosa* sp. library, 75 of 81 sequences were members of the phylum *Actinobacteria*, and the remaining clones were assigned to the phyla *Proteobacteria*, *Gemmatimonadetes*, and *Planctomycetes*. In the *Petrosia* sp. library, in addition to actinobacteria, sequences belonging to *Nitrospira* and *Gemmatimonadetes* were detected. No sequences other than *Actinobacteria* were detected in the *Cinachyra* sp. library. Comparison with the DDBJ also showed that the sequences from *Ulosa* sp. were the most diverse among the 3 sponges, sharing 17 different genera of actinobacteria as their closest neighbors. Because of low sequence similarity, assignment to the correct phylogenetic position became difficult. The RDP classifier program (34) assigned these sequences into 8 different genera (Table 1), and the remaining sequences were grouped as unclassified. Sequences sharing 10 different genera as their closest neighbors were detected in the actinobacteria library prepared from *Petrosia* sp., and members of only 4 genera were detected in the *Cinachyra* sp. library. Members of the family *Acidimicrobiaceae*, commonly found in association with sponges (22), were detected in all 3 sponge samples. In fact, sequences belonging to this family were dominant in *Cinachyra* sp. and *Petrosia* sp. Sequences assigned to the genus *Iamia*, a genus in the family *Iamiaceae*, was also detected in all 3 sponge samples. This family is also closely related to the family *Acidimicrobiaceae* and is a member of the same suborder, *Acidimicrobineae*.

MEGA (29) was used to calculate neighbor-joining and maximum parsimony trees. A phylogenetic tree based on 29, 22, and 8 representative sequences from *Ulosa* sp., *Petrosia* sp., and *Cinachyra* sp., respectively, is shown in Fig. 1. The tree can be divided into 2 clades, one of which is dominated by the sequences generally isolated from marine sponges. Most of the sequences obtained from *Cinachyra* sp. and *Petrosia* sp., along with the sponge-specific actinobacterial sequences described by Hentschel *et al.*(9), were grouped in this clade. Some sequences from *Petrosia* sp. form a stable branch, with a bootstrap value of 98%, with sponge-specific actinobacteria (9). Sequences in this clade belong to *Acidimicrobiaceae* and related families. Very few sequences from *Ulosa* sp. are grouped in this clade. The reasons for the lack of sponge-specific actinobacteria reported so far in *Ulosa* sp. are not known; *Ulosa* sp. may be an LMA sponge, the type generally considered to lack sponge-specific microbial communities. The second clade contains sequences from diverse actinobacterial families and mainly from *Ulosa* sp. Only 3 sequences from *Petrosia* sp. are grouped in this clade. Also, many sequences in these libraries, especially those from *Cinachyra* sp. and *Petrosia* sp., share very low sequence similarities with the sequences in DNA databanks (data not shown). This analysis clearly showed the presence of several novel actinobacteria in these sponge samples. Although strains belonging to 17 different genera were detected in *Ulosa* sp., 2 major groups represented the genera *Streptomyces* and *Actinomyces* and constituted 14% and 7%, respectively, of total clones. In *Petrosia* sp., the most dominant clones, which represented 50% of total clones, shared only 89–91% similarity with *Acidithiomicrobium* sp. (GQ225721). In *Cinachyra* sp., the most dominant clones, which constituted 70% of total clones, exhibited maximum similarity (89–92%) with *Ferrimicrobium* sp. (EU199234), which was thus its closest phylogenetic neighbor. Differences among the actinobacterial communities in the sponges studied seems not to depend on ambient seawater as all the sponge samples were collected from the same geographical location yet they harbored different microbial communities. This finding also indicates that the actinobacterial communities associated with sponges vary from sponge to sponge and may be sponge-specific.

We isolated actinobacteria from the sponges using 4 different culture media: starch-casein-nitrate agar (18), Gause 1 agar (2), manila clam (*Ruditapes philippinarum*) extract agar, and jewfish (*Argyrosomus argentatus*) extract agar (17). Jewfish and manila clam extract agars were used to provide complex (undefined) nutrients for bacterial growth. Isolated strains were maintained on ISP-2 medium [ISP-2M, ISP2; International *Streptomyces* project (25)] prepared in 50% seawater. Actinobacteria-like colonies with a powdery consistency were selected and spread on fresh ISP-2M until the cultures were pure. Interestingly, almost no actinobacterial colonies and only a few bacterial colonies appeared on the plates from *Petrosia* sp. and *Ulosa* sp. Based on colony morphology, only 13 strains were selected for further purification and 16S rRNA gene sequence analysis. Only 1 strain (PetSC-01) isolated from starch-casein-nitrate agar was closely related to *Micromonospora* sp. strain YIM 75717 (FJ911539), the only member of the phylum *Actinobacteria* isolated (Table 2). As observed in colony morphology, most strains were members of the phylum *Proteobacteria*. Notably, most of the strains isolated from *Petrosia* sp. and *Ulosa* sp. (9 of 13) exhibited maximum similarities with strains of marine origin. A list of strains isolated and their closest neighbors is provided in Table 2.

The failure to isolate any actinobacterial strain from *Petrosia* sp. and *Ulosa* sp. is interesting and may be because of the production of antimicrobial compounds by these 2 sponges. Therefore, we tested the antimicrobial activity of acetone extracts from these sponges against *Escherichia coli* W-3876, *Micrococcus luteus* ATCC 9341, *Candida albicans* NBRC 1594, and multiple *Streptomyces* strains (Table 2). Paper discs (6 mm in diameter) containing the acetone extracts from the sponges were placed on plates pre-seeded with the test organisms. The extract from *Ulosa* sp. exhibited strong antimicrobial activities against almost all of the test organisms. The extract from *Petrosia* sp. exhibited activity against *M. luteus* and a few strains of *Streptomyces*, possibly because of the low concentration of antimicrobial compounds produced by this sponge and/or insufficient extraction by acetone. Interestingly, all of the strains isolated from *Petrosia* sp. and *Ulosa* sp. were resistant or tolerant to the compounds produced by the respective sponges (data not shown). The extracts from *Petrosia* sp. and *Ulosa* sp. were analyzed for antibiotics by liquid chromatography–high-resolution mass spectrometry. *Ulosa* sp. was found to produce papuamine and its stereoisomer haliclonadiamine, pentacyclic alkaloids with antifungal and antibacterial activities. These compounds have been reported to be produced by the marine sponge *Haliclona* sp. (3, 33). *Petrosia* sp. produced some polyacetylene compounds with cytotoxic and antimicrobial activities, as described previously in the Introduction. These compounds may have killed microorganisms in the sponges, and the 16S rRNA gene sequences recovered may have originated from the dead cells. The possibility that these antimicrobial compounds are produced by bacteria associated with the sponges cannot be ruled out; therefore, the successful isolation of such actinobacteria from these sponges will be important in future studies. *Petrosia* sp. has been classified as an HMA sponge. Vacelet and Donadey (31) observed the presence of many bacterial morphotypes in *Petrosia ficiformis*; however, because these bacteria reside inside bacteriocytes and are possibly in symbiosis with sponge cells, isolation can be difficult. The clone sequences also show the dominance of *Acidimicrobiaceae*, which are notoriously difficult to culture.

Because *Cinachyra* sp. is dominated by members of the family *Acidimicrobiaceae*, the above-mentioned arguments may also apply to this sponge. In contrast, several actinobacterial colonies on all of the tested media appeared from *Cinachyra* sp. Based on colony morphology, 41 strains were isolated, and their partial 16S rRNA gene sequences were determined. Strains representing 10 different genera (*Actinomadura*, *Microbispora*, *Micromonospora*, *Nocardia*, *Nocardiopsis*, *Nonomuraea*, *Rhodococcus*, *Sphaerospo-rangium*, *Streptomyces*, and *Streptosporangium*) were isolated (data not shown). The most dominant genus was *Streptomyces* (51%), followed by *Rhodococcus* (12%) and *Micromonospora* (9%). These genera are also dominant among the actinobacteria isolated from other marine sponges (17, 37). The isolation medium is also an important factor; we isolated only 43 strains from *Cinachyra* sp. and found that the most diverse actinobacteria (6 different genera) were isolated from the manila clam extract agar designed in our previous study on *Haliclona* sp. (17). To the best of our knowledge, diverse actinobacteria from a single sponge have not been reported previously. Although members of 9 different genera were isolated from this sponge, an important finding is that none of the actinobacteria isolated were detected in the clone library including *Streptomyces*, which constitutes 51% of the isolated strains. This finding may be due to the bias of the PCR primers used. In our earlier studies on *Haliclona* sp. (17), we were unable to amplify any *Streptomyces* sequences using the set of primers used in this study (26); however, several *Streptomyces* sequences were amplified from the same DNA template when *Streptomyces*-specific primers (24) were used. Many of these clones even shared high similarity with the strains isolated from *Haliclona* sp. (17). Our results showed that *Cinachyra* sp. is a good source of actinobacteria and that these strains may be a prolific source of novel compounds, especially members of the genus *Streptomyces*. The results of our metabolite search of *Streptomyces* isolated from *Cinahyra* sp. identified the novel isoprenoids JBIR-46, JBIR-47, and JBIR-48 (14).

The 16S rRNA gene clone library analysis of these sponges suggested that actinobacterial communities associated with the studied sponges vary greatly despite sharing the same geographical location. *Cinachyra* sp. and *Petrosia* sp. are dominated by sponge-specific actinobacteria described by Hentschel *et al.*(9), but *Ulosa* sp. lacks such groups. These results suggest that *Ulosa* sp. is an LMA sponge, which lacks sponge-specific bacteria, and that *Cinachyra* sp. and *Petrosia* sp. are HMA sponges, which harbor sponge-specific actinobacteria. This theory is supported further by the isolation of diverse actinobacteria from *Cinachyra* sp. We speculated that the reason for our failure to isolate actinobacteria from *Ulosa* sp. and *Petrosia* sp. was the production of antimicrobial compounds; however, the presence of previously unreported novel actinobacteria in these sponges is evident from 16S rRNA gene clone libraries, and further attempts at isolation would be highly desirable. These actinobacteria may be a good source of novel bioactive compounds.

## Figures and Tables

**Fig. 1:**
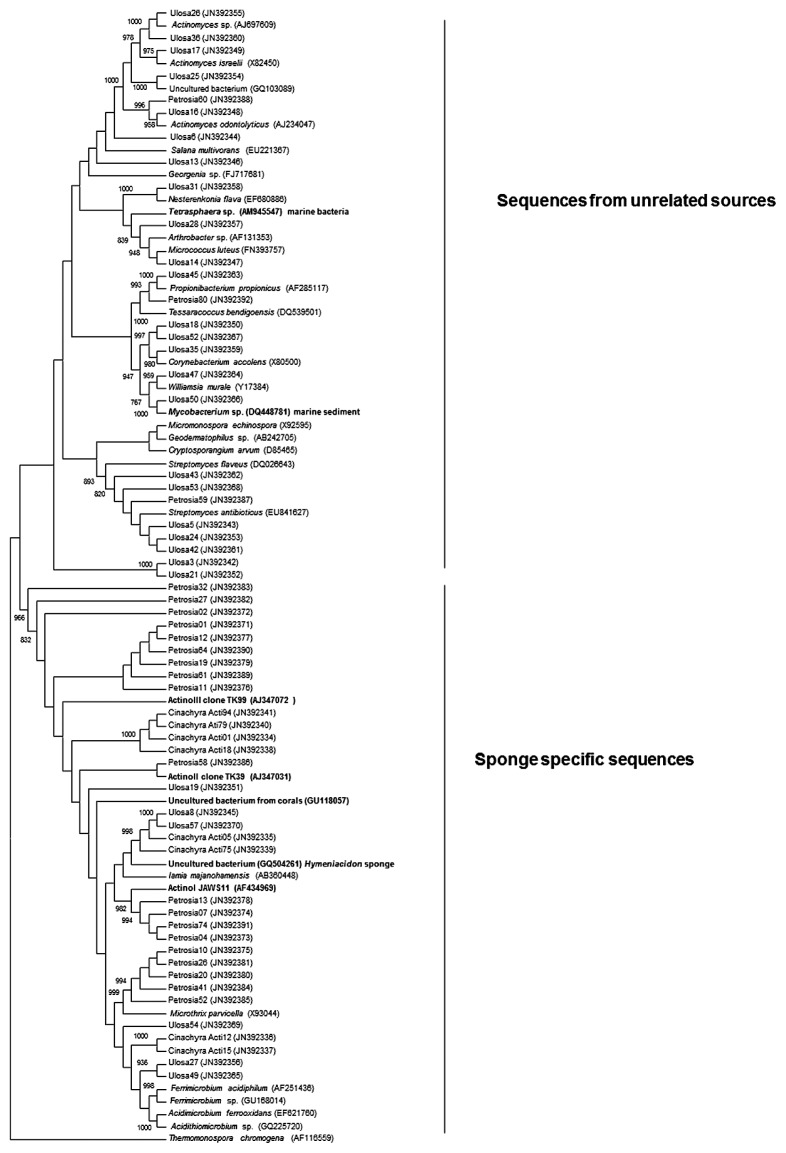
Phylogenetic dendrogram of representative actinobacteria clones from *Ulosa* sp., *Petrosia* sp., and *Cinachyra* sp. Related sequences were downloaded from the DNA Databank of Japan (DDBJ). The tree was calculated with a neighbor-joining algorithm and bootstrapped using 1000 replicates. Only those bootstrap values that exceeded 75% are shown.

**Table 1:** Actinobacterial diversity associated with three sponge samples collected from Ishigaki Island, Okinawa Prefecture, Japan. Genera detected in the sponge actinobacteria clone library were determined by BLAST and RDP classifier (Wang *et al.*, 2007*) analysis and biodiversity estimates

	Genera		
	
	*Ulosa* sp.	*Petrosia* sp.	*Cinachyra* sp.
Family			
*Acidimicrobiaceae*	*Ilumatobacter*	*Acidimicrobium*	Ilumatobacter*
	Ferrimicrobium*	Ferrimicrobium*	*Ferrimicrobium*
		Ferrithrix*	*Acidimicrobium*
*Acidothermaceae*	Acidothermus*		
*Actinomycetaceae*	Actinomyces*	*Actinomyces*	
*Beutenbergiaceae*	Salana*		
*Bogoriellaceae*	*Georgenia*		
*Corynebacteriaceae*	Corynebacterium*		
*Iamiaceae*	Iamia*	Iamia*	Iamia*
*Micrococcaceae*	*Arthrobacter*		
	Micrococcus*		
	*Rothia*		
	*Nesterenkonia*		
*Mycobacteriaceae*	*Mycobacterium*		
*Propionibacteriaceae*	*Propionibacterium*	*Propionibacterium*	
*Solirubrobacteraceae*		*Solirubrobacter*	
*Streptomycetaceae*	Streptomyces*	*Streptomyces*	
*Thermomonosporaceae*		*Thermomonospora*	
*“Williamsiaceae”*	*Williamsia*		
Unclassified	*Microthrix*	*Microthrix*	
Diversity estimates			
Total number of clones	81	63	93
No of clones after dereplication (OTUs)	58	48	5
Total number of genera detected	17	10	4
OTUs/Clone	0.71	0.76	0.05
Shannon–Wiener index (nats)	3.8863	3.6059	0.75
Simpson’s diversity index (1/D)	11.66	9.58	0.006

**Table 2:** Antimicrobial activity of the acetone extracts of the sponges against different strains

No.	Test strain	*Ulosa* sp.	*Petrosia* sp.
1	*Escherichia coli* W-3876	2.0	0
2	*Micrococcus luteus* ATCC 9341	2.5	1.0
3	*Candida albicans* NBRC 1594	1.0	0
4	*Streptomyces alanosinicus* NBRC 13493	2.0	0
5	*Streptomyces ambofaciens* ATCC 23877	1.5	0
6	*Streptomyces capoamus* NBRC 13411	2.3	0
7	*Streptomyces corchorusii* NBRC 13032	1.5	0
8	*Streptomyces cyaneus* NBRC 13346	1.9	0
9	*Streptomyces ferralitis* SFOp68	1.9	0
10	*Streptomyces glauciniger* NBRC 100913	1.5	0
11	*Streptomyces glomeratus* LMG 19903	0	0
12	*Streptomyces lanatus* NBRC 12787	2.1	0
13	*Streptomyces longwoodensis* LMG 20096	1.9	0
14	*Streptomyces mirabilis* NBRC 13450	1.9	0
15	*Streptomyces niveus* NBRC 12804	1.5	1.0
16	*Streptomyces olivoverticillatus* NBRC 15273	1.4	0
17	*Streptomyces paucisporeus* NBRC 102072	2.3	0.9
18	*Streptomyces psammoticus* NBRC 13971	1.3	0
19	*Streptomyces recifensis* NBRC 12813	0	0
20	*Streptomyces sanglieri* NBRC 100784	2.0	0
21	*Streptomyces speibonae* NBRC 101001	1.9	0
22	*Streptomyces yanglinensis* NBRC 102071	1.8	0

Zones of inhibition are given in cm.
